# Predictive value of bedside lung ultrasound, quantitative chest CT, and frailty assessment for short-term outcomes in elderly patients with severe pneumonia: a pilot study

**DOI:** 10.1186/s12890-025-03950-0

**Published:** 2025-10-15

**Authors:** Longjiang Shao, Yongyong Liang

**Affiliations:** Intensive Care Unit, The Fourth Affiliated Hospital of Soochow University, 9 Chongwen Road, Suzhou, Suzhou, Jiangsu 215000 China

**Keywords:** Elderly, Severe pneumonia, Lung ultrasound, Frailty, Pilot study

## Abstract

**Background:**

Elderly patients with severe community-acquired pneumonia (CAP) have high short-term mortality, yet conventional severity scores do not incorporate bedside imaging or physiological frailty. In this study we aim to (1) evaluate the feasibility of obtaining lung ultrasound (LUS), quantitative chest computed tomography (CT), and Clinical Frailty Scale (CFS) assessments during the same admission and (2) explore the predictive potential for 28-day mortality by integrating imaging severity with physiological frailty.

**Methods:**

In this prospective, single-center pilot study (February 2022 – February 2025), we consecutively enrolled 60 hospitalized adults ≥ 65 years who met guideline criteria for severe CAP and completed 28-day follow-up. Twelve-zone LUS and CFS assessments were performed ≤ 24 h after admission; chest CT was acquired within 48 h when clinically permissible. Feasibility outcomes were recruitment rate, data completeness, and study-related adverse events. Associations with 28-day mortality were analyzed descriptively and with exploratory multivariable logistic regression.

**Results:**

LUS and CFS were completed in 100% and 93.3% of participants, respectively; CT was obtained in 83.3%. No study-related adverse events occurred. Twelve patients (20%) died within 28 days. Compared with survivors, non-survivors had higher median LUS scores (14.1 vs. 12.1), greater CT-defined consolidation (30% vs. 22% of lung volume), and a higher prevalence of severe frailty (58% vs. 25%). In the 50 participants with complete data, both LUS score (odds ratio [OR] 1.09 per point) and severe frailty (OR 3.85) independently predicted mortality. A model combining LUS and frailty improved discrimination relative to CURB-65 alone (area under the receiver-operating characteristic curve 0.75 vs. 0.68).

**Conclusions:**

Simultaneous acquisition of LUS, quantitative CT, and frailty metrics is feasible, safe, and well tolerated in elderly patients with severe CAP. Preliminary evidence suggests that integrating imaging severity with physiological frailty enhances short-term risk stratification beyond established clinical scores. These findings merit confirmation in larger, multicenter cohorts.

**Supplementary Information:**

The online version contains supplementary material available at 10.1186/s12890-025-03950-0.

## Introduction

Severe pneumonia in older adults is a substantial public-health challenge, with an incidence roughly four times that observed in younger populations and correspondingly higher rates of hospitalization and mortality [1]. Although the Pneumonia Severity Index (PSI) and CURB-65 remain the most widely used prognostic tools, their accuracy in geriatric care is limited. These scores were derived primarily from younger cohorts and do not account for age-specific factors such as baseline frailty, multimorbidity, and the atypical symptom profiles frequently occur in elderly patients [2, 3].

Chest computed tomography (CT) provides high-sensitivity, quantitative assessment of pulmonary involvement but is constrained by ionizing-radiation exposure and the logistical challenges of transporting hemodynamically unstable patients [4, 5]. In contrast, bedside lung ultrasound (LUS) is non-invasive, repeatable, and radiation-free [6, 7]; its diagnostic accuracy for pneumonia is comparable to that of CT [8, 9] and is increasingly valued in critical-care settings for real-time therapeutic guidance and monitoring of disease progression [7, 10]. However, the prognostic utility of LUS in severe pneumonia remains uncertain: although it readily identifies B-lines, dynamic air bronchograms, and pleural effusions, its ability to predict clinical outcomes has not been firmly established [6, 11]. Integrating LUS with CT can enhance diagnostic precision and refine severity assessment [4], yet further studies are needed to delineate how LUS-derived metrics can be incorporated into robust prognostic models.

Frailty—characterized by a decline in physiological reserve and impaired homeostatic mechanisms—has become a robust, independent predictor of poor outcomes in older adults [12, 13]. It consistently forecasts adverse events across diverse clinical settings, including infectious diseases, cardiovascular pathology, and surgical procedures [14, 15]. In community-acquired pneumonia (CAP), higher frailty scores are associated with greater in-hospital complications and mortality [16]. Because frailty captures vulnerability beyond chronological age and comorbidity burden [17], integrating it into pneumonia severity algorithms holds considerable promise. Nevertheless, studies that combine frailty metrics with imaging-based assessments remain scarce [16]. Given the independent prognostic weight of frailty in CAP and other acute illnesses [16, 18], systematic incorporation of frailty evaluations could meaningfully refine risk stratification and inform personalized management strategies for elderly patients [18, 19].

Accordingly, we undertook a single-center pilot study with three objectives: (i) to establish the feasibility—quantified by recruitment rate, data completeness, and safety—of collecting LUS, quantitative CT, and Clinical Frailty Scale (CFS) data within 48 h of admission in adults aged ≥ 65 years with severe pneumonia; (ii) to evaluate the independent and combined prognostic value of these metrics for 28-day mortality; and (iii) to delineate logistical challenges that should inform the design of a subsequent multicenter trial.

## Methods

### Study design and setting

This prospective, single-center pilot study was conducted at the Fourth Affiliated Hospital of Soochow University from February 2022 to February 2025. We consecutively screened all adults ≥ 65 years admitted with radiologically confirmed CAP. The study protocol was approved by the hospital’s Institutional Review Board, and written informed consent was obtained from each participant or, when decision-making capacity was impaired, from an authorized surrogate. All procedures adhered to the Declaration of Helsinki. Patients (or their surrogates) received detailed explanations of the study aims and procedures. To limit therapeutic bias, treating clinicians were blinded to lung-ultrasound findings unless an immediate clinical intervention was deemed necessary, in which case partial unblinding was permitted.

*Inclusion Criteria: *1), Age ≥ 65 years; 2), Radiologically confirmed pneumonia. A new or progressive pulmonary infiltrate visible on chest radiograph or computed-tomography (CT) obtained within 48 h of presentation, and at least one supportive clinical feature (fever, leukocytosis or leukopenia, purulent sputum, or elevated inflammatory markers). Bedside lung-ultrasound findings alone were not accepted because they are not formally reported or archived in the hospital’s imaging system; 3), Severe pneumonia (Defined by fulfilment of ≥ 1 of the following: CURB-65 ≥ 3; PSI class IV or V; or intensive-care-unit admission for ventilatory or vasopressor support; 4), Community-acquired infection. Symptom onset and/or first positive imaging occurring ≤ 48 h after hospital arrival, thereby excluding hospital-acquired pneumonia. Only patients satisfying all four criteria were enrolled and subjected to study procedures.

*Exclusion criteria:* 1), Inability to obtain informed consent. Refusal or incapacity to provide written consent, with no legally authorized surrogate available; 2), Hemodynamic instability precluding safe CT transport. Patients too unstable for the radiology suite were excluded from the CT component but could still undergo bedside LUS and frailty assessment to maximize data capture; 3), End-stage comorbidity with limited life expectancy. Anticipated survival < 1 month from a condition unrelated to pneumonia (e.g., metastatic malignancy receiving palliative care); 4), Hospital-acquired or ventilator-associated pneumonia. Infection onset ≥ 48 h after hospital admission or ≥ 48 h after endotracheal intubation, respectively. Poor acoustic windows on the initial LUS examination did not constitute an exclusion criterion; sonographers were instructed to adjust probe position, frequency, or patient posture to obtain interpretable images whenever possible.

#### Sample size calculation

Given the pilot nature of the study and the expected recruitment capacity over the 36-month enrolment window, we targeted a cohort of 60 participants. This number was selected primarily to evaluate operational feasibility—specifically, screening efficiency, data-capture completeness, and safety—and to generate preliminary effect-size estimates for key outcomes. These estimates will underpin formal sample-size calculations for a subsequent, adequately powered multicenter trial.

Partial unblinding was prospectively defined as any instance in which the study sonographer or interpreting radiologist identified an imaging abnormality requiring immediate clinical intervention (e.g., an unsuspected large pleural effusion or pneumothorax) and therefore disclosed the finding to the treating team before database lock. Every such episode was to be prospectively logged and subsequently detailed in the Results section.

### Study procedures

Severe pneumonia was defined when any of the following criteria were met: (i) a CURB-65 score ≥ 3; (ii) PSI class IV or V; or (iii) immediate need for intensive-care–level organ support (invasive or non-invasive mechanical ventilation, or vasopressor therapy). For each enrollee, we prospectively documented the specific criterion—or combination of criteria—by which severe disease was established.

Bedside LUS examinations were completed within 24 h of admission by either a critical-care physician or a registered sonographer, each certified according to International Consensus Conference on Lung Ultrasound guidelines. Scans were acquired with a portable ultrasound unit (Mindray M9; Mindray Bio-Medical, Shenzhen, China). The default transducer was a 2- to 5 MHz curvilinear probe; a 5- to 12 MHz linear probe was substituted when higher-resolution pleural imaging or improved penetration through subcutaneous emphysema was necessary.

A standardized 12-zone protocol—dividing each hemithorax into upper and lower anterior, lateral, and posterior regions—was used. Each zone was systematically assessed for B-lines, subpleural consolidations, and pleural effusions. Findings were summarized in a semi-quantitative LUS score (0–24), reflecting the number and extent of abnormalities across all zones. To evaluate inter-observer reliability, a random sample of 15 patients underwent repeat scanning by a second blinded examiner within 1 h of the initial assessment.

#### Quantitative chest CT

Chest CT was performed within 48 h of admission provided the patient’s hemodynamic status permitted safe transport to the radiology suite. All scans were acquired on a 320-detector-row scanner (Aquilion ONE, Canon Medical Systems, Otawara, Japan) using a low-dose protocol (collimation 1 mm; tube current 80–120 mA with automated dose modulation). Non-contrast, low-dose CT constituted the default protocol; contrast-enhanced CT angiography was reserved for specific clinical indications—namely suspected pulmonary embolism, complicated pleural effusion, or mediastinal infection—at the discretion of the attending physician.

A radiographer uploaded the images to dedicated post-processing software equipped with Advanced Clear-IQ Engine (Canon Medical Systems). Using fully automated or semi-automated segmentation, the software quantified: (i) the percentage of lung volume occupied by consolidation and (ii) the number of lobes involved. Incidental thoracic findings (e.g., emphysematous changes, pulmonary nodules) were recorded prospectively. All quantitative outputs were exported to the study database for subsequent analysis.

#### Frailty assessment

Frailty was evaluated with the CFS, which grades functional status from 1 (very fit) to 9 (terminally ill). A trained geriatric nurse practitioner or physician completed each assessment within 24–48 h of admission, drawing on the pre-morbid history, physical examination, and baseline functional capacity. For analytic purposes, CFS scores were grouped as mild frailty (4–5), moderate frailty (6), and severe frailty (7–8); patients with end-of-life status (CFS 9) were excluded per protocol.

#### Feasibility end‑points

We prospectively captured three metrics to assess feasibility: (i) recruitment efficiency, calculated as the proportion of screened patients who were successfully enrolled; (ii) data completeness for the three key assessments—LUS, chest CT, and CFS; and (iii) safety, defined as any study-related adverse event (e.g., oxygen desaturation during LUS or hemodynamic instability during CT transport). A screening log recorded all reasons for non-enrolment and documented logistical barriers encountered during study procedures.

### Statistical analysis

Continuous variables are expressed as mean ± standard deviation (SD) when normally distributed, or as median [interquartile range, IQR] when non-normally distributed. Group differences were evaluated with Student’s t-test or the Mann–Whitney U test, as appropriate. Categorical variables are presented as counts (percentages) and compared using the χ² test or Fisher’s exact test. Because of the limited sample size, a parsimonious multivariable logistic-regression model was constructed for 28-day mortality, incorporating prespecified covariates with established clinical relevance (LUS score, percentage lung consolidation on CT, frailty category, and age). All analyses were performed in R (version 4.4). Given the pilot nature of the study, no adjustments were made for multiple comparisons. Two-tailed p values < 0.05 were considered statistically significant.

## Results

**Fig. 1:**
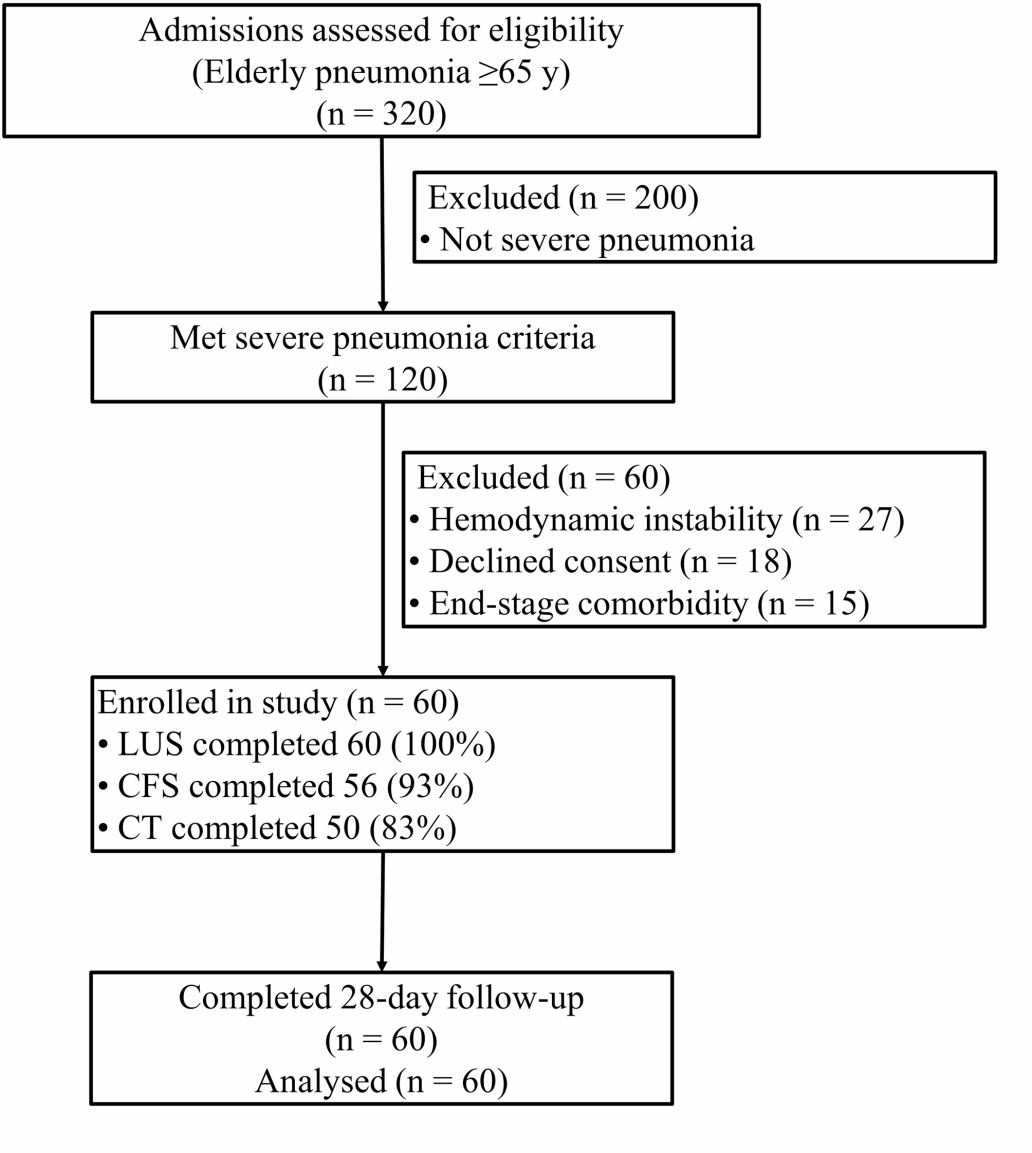
Flowchart of participant enrollment

Between February 2022 and February 2025, the ICU admitted 320 patients aged ≥ 65 years with radiologically confirmed pneumonia (Fig. 1); 120 fulfilled at least one predefined severe-pneumonia criterion. After screening, 60 patients were excluded. LUS was completed in all 60 patients, the CFS in 56, and chest CT in 50, with no study-related adverse events.

The cohort’s mean age was 78.3 ± 7.9 years, and slightly more than half were male (Table 1). Hypertension and diabetes were the most common comorbidities. Disease severity was substantial: the mean CURB-65 score was 3.2 ± 0.8, and 63.3% of participants were classified as PSI class IV or V (Table 1). Among the 56 patients assessed for frailty, 33.9% were severely frail (CFS 7–8). Thirty-six patients (60%) were admitted directly to the ICU, whereas 24 (40%) began treatment on a high-dependency respiratory ward; all satisfied ≥ 1 severe-pneumonia criterion. Intravenous contrast was administered in 8 of 50 CT scans (16%): five for pulmonary-embolism work-up, two for pleural-space evaluation, and one for suspected mediastinal abscess.

**Table 1:** Baseline demographics and clinical characteristics

	Value
Age (years), mean ± SD	78.3 ± 7.9
Male, n (%)	35 (58.3%)
Hypertension, n (%)	31 (51.7%)
Diabetes mellitus, n (%)	18 (30.0%)
COPD, n (% of total)	12 (20.0%)
CKD (Stage ≥ 3), n (%)	9 (15.0%)
CURB-65 Score, mean ± SD	3.2 ± 0.8
PSI Class IV or V, n (%)	38 (63.3%)
Pathogen category, n (%)	
* Streptococcus pneumoniae*	12 (20.0%)
* Haemophilus influenzae*	4 (6.7%)
Other typical bacteria	3 (5.0%)
Atypical bacteria	5 (8.3%)
Influenza A/B	6 (10.0%)
RSV	2 (3.3%)
SARS‑CoV‑2	4 (6.7%)
Coinfection (any bacterial + viral)	5 (8.3%)
No pathogen identified	24 (40.0%)
Frailty (CFS) completed, n (%)	56 (93.3%)
– Mild Frailty (CFS 4–5)	17 (30.4% of those assessed)
– Moderate Frailty (CFS 6)	20 (35.7% of those assessed)
– Severe Frailty (CFS 7–8)	19 (33.9% of those assessed)
Initial location, n (%)	
ICU	36 (60.0%)
Respiratory HDU	24 (40.0%)
Contrast CT	8 (16.0%)

All 60 participants met ≥ 1 severe-pneumonia criterion (Supplementary Table S1). Specifically, 47 had a CURB-65 score ≥ 3, 38 were PSI class IV or V, and 42 required ICU-level organ support on admission; 27 satisfied all three. Complete LUS, CT, and CFS data were available for 50 patients (83%); the remaining 10 lacked CT (transport instability) and/or CFS (no surrogate informant). Age, sex, severity scores, comorbidity burden, and 28-day mortality did not differ between complete-case and missing-data cohorts (all *p* > 0.10; Supplementary Table S2). Little’s MCAR test supported a “missing completely at random” pattern (χ² = 5.2, df = 6, *p* = 0.52).

Twelve patients (20%) died within 28 days (Table 2). Non-survivors had higher mean LUS scores and more extensive CT consolidation than survivors (*p* = 0.031), and severe frailty (CFS ≥ 7) was more prevalent among non-survivors (Table 2). Age and sex distributions were similar between groups.

**Table 2:** Comparison of selected variables in survivors vs. Non-Survivors

	Survivors (*n* = 48)	Non-Survivors (*n* = 12)	*p*-Value
Age (years), mean ± SD	77.9 ± 8.0	80.0 ± 7.4	0.40
Male, n (%)	28 (58.3%)	7 (58.3%)	0.99
LUS Score, mean ± SD	12.1 ± 3.9	14.1 ± 3.9	0.045
CT Consolidation (% lung volume), median (IQR)	22% (IQR 15–28%)	30% (IQR 25–38%)	0.031
Severe Frailty (CFS 7–8), n (%)	12 (25.0%)	7 (58.3%)	0.015
CURB-65 ≥ 3, n (%)	30 (62.5%)	10 (83.3%)	0.19

Inter-rater reliability for LUS was high: in 15 randomly selected patients, Cohen’s kappa for detecting subpleural consolidations was 0.82 (95% CI 0.68–0.96), and the intraclass correlation coefficient for total LUS score was 0.85 (95% CI 0.75–0.92) (Table 3).

**Table 3:** Inter-Rater reliability of key LUS parameters in 15 randomly selected patients

Parameter	Measure of Agreement	95% CI
Presence of B-lines (yes/no)	Cohen’s kappa = 0.78	0.63–0.93
Subpleural Consolidations	Cohen’s kappa = 0.82	0.68–0.96
LUS Score (continuous)	ICC (2,1) = 0.85	0.75–0.92

Mortality rose with increasing frailty, reaching 36.8% in severely frail patients versus 11.8% in those mildly frail (*p* = 0.015; Table 4). ICU length of stay followed the same pattern (median 12 [IQR 8–16] days vs. 6 [4–7] days).

**Table 4:** Outcomes stratified by frailty category (CFS)

Frailty Category	Number of Patients	28-Day Mortality, *n* (%)	Median ICU LOS (IQR)	Mechanical Ventilation, *n* (%)
Mild (CFS 4–5)	17	2 (11.8%)	6 (4–7) days	6 (35.3%)
Moderate (CFS 6)	20	3 (15.0%)	8 (5–10) days	9 (45.0%)
Severe (CFS 7–8)	19	7 (36.8%)	12 (8–16) days	13 (68.4%)
p-Value	—	0.015	0.002	0.06

In an exploratory logistic-regression model (*n* = 50), higher LUS scores and severe frailty were independently associated with 28-day mortality (Table 5); CT consolidation showed a borderline association (OR = 1.02, 95% CI 1.00–1.05, *p* = 0.058), whereas age was not significant. Model diagnostics revealed no influential outliers (Cook’s distance < 0.2) and acceptable collinearity (maximum VIF = 2.1). Firth-penalized estimates were comparable (Supplementary Table S3), indicating limited over-fitting.

**Table 5:** Exploratory logistic regression for 28-Day mortality

	Odds Ratio	95% CI	*p*-Value
LUS Score (per + 1)	1.09	1.01–1.19	0.027
CT Consolidation (%)	1.02	1.00–1.05	0.058
Severe Frailty (CFS ≥ 7)	3.85	1.11–13.3	0.034
Age (per year)	1.02	0.96–1.09	0.52
Constant	—	—	0.003

Complete-case sample *n* = 50; events (deaths) = 12

For mortality prediction (*n* = 50), CURB-65 alone yielded an AUROC of 0.68 (95% CI 0.53–0.83; Table 6). A LUS score ≥ 13 provided an AUROC of 0.70 (0.56–0.85; *p* = 0.40 vs. CURB-65), CT consolidation ≥ 28% an AUROC of 0.69 (0.54–0.84; *p* = 0.46), and severe frailty an AUROC of 0.72 (0.59–0.85; *p* = 0.24). Combining LUS with frailty improved the AUROC to 0.75 (0.62–0.87), and adding CT raised it to 0.77 (0.63–0.88); neither composite exceeded CURB-65 at conventional significance levels (*p* = 0.10 and 0.06, respectively).

**Table 6:** ROC curve analysis: comparison with standard severity scores

Model/Predictor	AUC (95% CI)	*p* vs. CURB-65
CURB-65	0.68 (0.53–0.83)	—
LUS total score (≥ 13)	0.70 (0.56–0.85)	0.40
CT % consolidation (≥ 28%)	0.69 (0.54–0.84)	0.46
Severe frailty (CFS ≥ 7)	0.72 (0.59–0.85)	0.24
LUS + Frailty	0.75 (0.62–0.87)	0.10
LUS + CT + Frailty	0.77 (0.63–0.88)	0.06

## Discussion

Our pilot data demonstrate that bedside lung ultrasound, quantitative low-dose chest CT, and Clinical Frailty Scale assessment can be integrated seamlessly within the first 48 h of admission in older adults with severe CAP. The high rates of data completeness and absence of study-related adverse events indicate that a larger, multicenter trial is both feasible and acceptable to patients. Clinically, higher LUS and CT consolidation scores—together with greater frailty—were each independently associated with 28-day mortality. These findings support a multimodal “imaging-frailty” strategy to sharpen early risk stratification and guide individualized management in this vulnerable population.

Our pilot data underscore the emerging consensus that pairing bedside imaging with a frailty framework yields a more nuanced risk profile in older adults with community-acquired pneumonia. In our 60-patient cohort, each one-point increase in the 12-zone LUS aeration score was associated with an 8% rise in the odds of 28-day mortality, and non-survivors displayed substantially higher median scores than survivors. These observations corroborate earlier evidence that LUS surpasses chest radiography for pneumonia detection [20] and closely reflects CT-quantified parenchymal involvement [21–23]. Quantitative low-dose CT provided complementary anatomic detail: patients with >28% consolidated lung volume experienced nearly a two-fold increase in mortality risk, although routine use remains limited by transport logistics and hemodynamic concerns [20, 24]. Frailty added an independent biological dimension—patients with a CFS score ≥ 7 faced a four-fold higher risk of death [25, 26]. Integrating LUS, CT consolidation, and severe frailty in a parsimonious multivariable model improved discrimination from 0.68 with CURB-65 alone to 0.77, with re-classification analyses indicating that approximately one in five patients would be appropriately shifted to a higher-risk stratum. Collectively, these findings suggest that real-time lung imaging combined with frailty assessment offers modest but clinically meaningful gains in prognostication beyond traditional physiology-based scores [23, 27].

Pathophysiologically, frailty and extensive pulmonary involvement appears to synergistically worsen outcomes in elderly patients with CAP. Chronic low-grade inflammation and diminished physiological reserve—the hallmarks of frailty—may amplify the cytokine surge triggered by severe infection, thereby helping to explain the independent association between frailty and higher CAP-related mortality [25, 26, 28–31]. Radiological indicators such as large consolidations or diffuse B-lines probably reflect a greater pathogen burden or more aggressive host response, further compounding vulnerability in this population. Although heterogeneity in frailty definitions and severity criteria for pneumonia may contribute to variability across studies [2, 32], the consistent links observed in our cohort and prior research underscore the need to consider both frailty status and imaging severity when stratifying risk and tailoring management in older adults with CAP.

This single-center pilot demonstrates that a streamlined bedside bundle—comprising a 12-zone LUS examination, low-dose quantitative chest CT, and same-day CFS assessment—can be completed within 48 h in older adults with severe CAP. LUS and CFS were obtained in 100% and 93% of participants, respectively, and CT in 83%, with no procedure-related complications. These feasibility metrics parallel the practicality of the BLUE protocol, which achieves 88% sensitivity and 90% specificity for acute respiratory failure when employed by non-expert operators [33, 34], and they extend previous work by showing that the Pneumonia LUS Score and frailty assessment can be integrated without delaying clinical care [35, 36]. Consistent with prior studies linking LUS aeration scores to CT-derived lung involvement in viral pneumonias [37–39], higher LUS values in our cohort correlated with increased 28-day mortality, while CT-quantified consolidation offered complementary anatomical detail. When imaging metrics were combined with severe frailty (CFS ≥ 7), the composite model (AUROC 0.77) outperformed CURB-65 alone (0.68), reinforcing the premise that real-time lung imaging, augmented by a frailty lens, meaningfully enhances traditional severity indices in elderly CAP.

Several operational barriers identified in this pilot must be addressed before a multicenter roll-out. First, 16% of participants required contrast-enhanced CT to rule out pulmonary embolism or empyema. The associated ICU-to-scanner transfers mirrored the safety challenges reported in SARS-CoV-2 related ARDS, where transport-induced delirium and agitation were common [40]. Second, recruitment was slower than anticipated because many frail geriatric candidates presented with delirium, complicating consent procedures [41–43]. Third, providing round-the-clock LUS strained existing staffing levels; similar training and personnel demands have been documented during continuous ultrasound monitoring in influenza-A cohorts [40]. Finally, harmonizing image archiving across different devices proved difficult—a long-standing obstacle to data integrity in multicenter ventilator-associated-pneumonia studies [44–46].

Mitigation strategies for future trials should therefore include portable CT units or contrast-avoidance algorithms, streamlined consent pathways for patients with cognitive impairment, dedicated ultrasound staffing or tele-mentoring models, and unified, interoperable imaging-data platforms. Tackling these logistical hurdles will be crucial to the successful scaling of this multimodal imaging-frailty approach.

Despite its strengths, this pilot study has important limitations that temper the generalizability of its findings. First, the modest sample size and single-center design introduce potential selection bias and restrict external validity. Recruitment lagged chiefly because nearly half of screened candidates were too unstable for CT transport and one-fifth declined consent. Second, frailty assessment occasionally relied on proxy informants, prolonging enrolment and contributing to incomplete data. These hurdles echo those reported in prior geriatric-pneumonia investigations and highlight operational requirements for future studies—namely, (i) round-the-clock research staffing, (ii) portable low-dose CT or LUS-only pathways when transport is unsafe, and (iii) expedited surrogate-consent procedures.

Given these constraints, our convenience sample may not fully represent the broader elderly population with severe CAP. Future research should therefore validate these preliminary observations in larger, multicenter cohorts; refine predictive models that integrate imaging and frailty metrics; and extend follow-up to capture 90-day mortality, functional recovery, and healthcare utilization. Cost-effectiveness analyses are also warranted to ensure that resource-intensive investigations are applied judiciously in this vulnerable group.

## Conclusion

This pilot study demonstrates that a streamlined, multimodal assessment—combining bedside lung ultrasound, low-dose quantitative chest CT, and Clinical Frailty Scale scoring—can be performed within 48 h of admission in elderly patients with severe community-acquired pneumonia. Each component provided independent prognostic information, and their integration modestly but meaningfully improved short-term risk discrimination beyond CURB-65. Although validation in larger, multicenter cohorts is needed, our findings highlight the potential clinical value of pairing imaging severity with frailty status to refine prognosis and support more individualized management in this vulnerable population.

## Supplementary Information

Supplementary Material 1.

## Data Availability

Data sets generated during the current study are available from the corresponding author on reasonable request.

## Abbreviations

CAP: Community-acquired pneumonia
CFS: Clinical Frailty Scale
CKD: Chronic kidney disease
COPD: Chronic obstructive pulmonary disease
CT: Computed tomography
CURB-65: Confusion, Urea, Respiratory rate, Blood pressure, Age ≥65 score
ICU: Intensive-care unit
IQR: Interquartile range
LUS: Lung ultrasound
PSI: Pneumonia Severity Index
SD: Standard deviation
