# Supplementary material for: Predictive value of bedside lung ultrasound, quantitative chest CT, and frailty assessment for short-term outcomes in elderly patients with severe pneumonia: a pilot study
Source: BMC Pulm Med. 2025 Oct 15;25:474. doi: 10.1186/s12890-025-03950-0 (PMC12522310; doi:10.1186/s12890-025-03950-0)
Supplement: Supplementary file 1 — Supplementary Material 1. [file 12890_2025_3950_MOESM1_ESM.docx]

**Supplementary Table S1. Classification of participants**

| **Severe‑pneumonia criterion** | **n (%) of 60 patients** |
| --- | --- |
| CURB‑65 ≥ 3 | **47 (78.3%)** |
| PSI class IV / V | **38 (63.3%)** |
| ICU‑level organ support on admission | **42 (70.0%)** |
| All three criteria met | **27 (45.0%)** |

**Supplementary Table S2. Baseline characteristics by data‑completeness status**

| **Variable** | **Complete‑case cohort (n = 50)** | **Missing‑data cohort (n = 10)** | **p‑value*** |
| --- | --- | --- | --- |
| Age, y (mean ± SD) | 78 ± 6 | 79 ± 5 | 0.59 |
| Male sex, n (%) | 29 (58.0%) | 6 (60.0%) | 0.88 |
| CURB‑65 ≥ 3, n (%) | 40 (80.0%) | 8 (80.0%) | 1.00 |
| PSI class IV/V, n (%) | 31 (62.0%) | 7 (70.0%) | 0.62 |
| Comorbidity count, median (IQR) | 3 (2 – 4) | 3 (2 – 4) | 0.97 |
| 28‑day mortality, n (%) | 12 (24) | 0 (0) | 0.11 |

* Student’s t‑test for continuous variables (age), χ² or Fisher’s exact test for categorical variables, Mann‑Whitney U for counts

**Supplementary Table S3. Firth‑penalized logistic regression for 28‑day mortality after multiple imputation**

| **Predictor (entered simultaneously)** | **β (log‑odds)** | **SE** | **Odds ratio** | **95% CI for OR** | **p‑value†** |
| --- | --- | --- | --- | --- | --- |
| LUS score (per +1 point) | 0.075 | 0.032 | **1.08** | 1.01 – 1.17 | 0.026 |
| CT consolidation (% lung; per +1%) | 0.019 | 0.010 | **1.02** | 1.00 – 1.05 | 0.055 |
| Severe frailty (CFS ≥ 7 vs <7) | 1.28 | 0.64 | **3.60** | 1.05 – 12.4 | 0.041 |
| Age (per +1 year) | 0.015 | 0.031 | 1.02 | 0.96 – 1.09 | 0.51 |
| **Intercept** | −9.42 | 4.01 | — | — | 0.016 |

Model diagnostics: AUC = 0.76; maximum VIF = 2.1; no influential observations (Cook’s distance < 0.2).
